# Probiotic *Bifidobacterium* strains and galactooligosaccharides improve intestinal barrier function in obese adults but show no synergism when used together as synbiotics

**DOI:** 10.1186/s40168-018-0494-4

**Published:** 2018-06-28

**Authors:** Janina A. Krumbeck, Heather E. Rasmussen, Robert W. Hutkins, Jennifer Clarke, Krista Shawron, Ali Keshavarzian, Jens Walter

**Affiliations:** 1 Department of Food Science and Technology, 258 Food Innovation Center Lincoln, University of Nebraska-Lincoln, Lincoln, NE 68588 USA; 2 Department of Clinical Nutrition, Rush University Medical Center, Chicago, IL USA; 3 Division of Digestive Diseases and Nutrition, Department of Internal Medicine, Rush University Medical Center, Chicago, IL USA; 4 Department of Biological Sciences, University of Alberta, Edmonton, AB Canada; 5 Department of Agricultural, Food, and Nutritional Science, University of Alberta, Edmonton, AB Canada; 6 4-126A Li Ka Shing Centre for Health Research Innovation and 7-142 Katz Group Center, Edmonton, AB T6G 2E1 Canada

**Keywords:** Synbiotic, Probiotic, Prebiotic, Obesity, Gut barrier function, Autochthonous, Allochthonous, Galactooligosaccharide, Bifidobacteria, *Bifidobacterium*

## Abstract

**Background:**

One way to improve both the ecological performance and functionality of probiotic bacteria is by combining them with a prebiotic in the form of a synbiotic. However, the degree to which such synbiotic formulations improve probiotic strain functionality in humans has not been tested systematically. Our goal was to use a randomized, double-blind, placebo-controlled, parallel-arm clinical trial in obese humans to compare the ecological and physiological impact of the prebiotic galactooligosaccharides (GOS) and the probiotic strains *Bifidobacterium adolescentis* IVS-1 (autochthonous and selected via in vivo selection) and *Bifidobacterium lactis* BB-12 (commercial probiotic allochthonous to the human gut) when used on their own or as synbiotic combinations. After 3 weeks of consumption, strain-specific quantitative real-time PCR and 16S rRNA gene sequencing were performed on fecal samples to assess changes in the microbiota. Intestinal permeability was determined by measuring sugar recovery in urine by GC after consumption of a sugar mixture. Serum-based endotoxin exposure was also assessed.

**Results:**

IVS-1 reached significantly higher cell numbers in fecal samples than BB-12 (*P* < 0.01) and, remarkably, its administration induced an increase in total bifidobacteria that was comparable to that of GOS. Although GOS showed a clear bifidogenic effect on the resident gut microbiota, both probiotic strains showed only a non-significant trend of higher fecal cell numbers when administered with GOS. Post-aspirin sucralose:lactulose ratios were reduced in groups IVS-1 (*P* = 0.050), IVS-1 + GOS (*P* = 0.022), and GOS (*P* = 0.010), while sucralose excretion was reduced with BB-12 (*P* = 0.002) and GOS (*P* = 0.020), indicating improvements in colonic permeability but no synergistic effects. No changes in markers of endotoxemia were observed.

**Conclusion:**

This study demonstrated that “autochthony” of the probiotic strain has a larger effect on ecological performance than the provision of a prebiotic substrate, likely due to competitive interactions with members of the resident microbiota. Although the synbiotic combinations tested in this study did not demonstrate functional synergism, our findings clearly showed that the pro- and prebiotic components by themselves improved markers of colonic permeability, providing a rational for their use in pathologies with an underlying leakiness of the gut.

**Electronic supplementary material:**

The online version of this article (10.1186/s40168-018-0494-4) contains supplementary material, which is available to authorized users.

## Background

It is now well established that the gastrointestinal (GI) microbiota has a fundamental impact on metabolic, immunological, and endocrine functions of the host [1–4]. The host-microbe interrelationship is viewed as mutualistic, contributing to overall host health [5]. However, aberrations in both microbiota composition and function can result in the development of several chronic disease states [6–8]. Obesity, in particular, is associated with a dysbiosis characterized by low diversity and specific shifts in bacterial taxa that correlate with metabolic and inflammatory markers [9]. Although causation has not yet been established in humans, these associations suggest that gut microbes contribute to the subclinical systemic inflammation that ultimately lead to the development of pathologies such as insulin resistance, type-2 diabetes, and cardiovascular disease [10, 11]. One mechanism by which the gut microbiota contributes to systemic inflammation is through the translocation of pro-inflammatory and immunogenic bacterial compounds, including lipopolysaccharide (LPS) and peptidoglycan, that can drive metabolic endotoxemia [12, 13]. In fact, intestinal permeability has been shown to be elevated in at least a subset of obese subjects and is increased by high-fat diets, potentially constituting a mechanism by which the microbiota contribute to obesity [14]. The microbiome’s influence on both the development of endotoxemia as well as intestinal barrier integrity makes it a rational target for dietary strategies aimed at reducing intestinal epithelial permeability and obesity-associated pathologies.

Data from animal and in vitro studies suggest that both specific bacterial taxa and their metabolic products influence intestinal barrier function. For example, short-chain fatty acids produced from the fermentation of non-digestible carbohydrates (including commonly used prebiotics) have been reported to improve expression of tight junction proteins, such as zona occludens proteins (ZO-1-2), occluding, and claudin 1-4 (CLDN1-4) [15–19]. In addition, several studies suggest that probiotics, including *Bifidobacterium* spp., *Lactobacillus* spp., and other lactic acid bacteria, were associated with barrier function integrity in vitro and in vivo [20–25]. In addition to strong associations between *Bifidobacterium* numbers and improvements in intestinal epithelial cell barrier function and intestinal permeability [12, 26, 27], functional studies have begun to establish a causative role. For example, a strain of *Bifidobacterium longum* subsp. *infantis* (recently reclassified as *Bifidobacterium animalis* subsp. *lactis* [28]) increased trans-epithelial resistance and expression of tight junction proteins in IL-10-deficient mice [29] and decreased intestinal permeability in mice suffering from necrotizing enterocolitis [30]. Furthermore, treatment with *B*. *infantis* and *Bifidobacterium bifidum* decreased the gut endotoxin concentration in mice [31], and *Bifidobacterium adolescentis* administration to rats significantly lowered rates of bacterial translocation [32]. Bifidobacteria have also been associated with metabolic improvements considered to be associated with inflammation, including insulin sensitivity, white fat accumulation, liver weight [33], reactive oxygen species, nuclear factor κB activation, and reduced markers of inflammation [34], and high-density lipoprotein (HDL) plasma cholesterol levels [35].

These findings provide a rational basis for the development of strategies intended to enrich for *Bifidobacterium* populations in the human gut. This can be achieved through dietary consumption of probiotics and prebiotics. The consumption of prebiotic carbohydrates, such as galactooligosaccharide (GOS), resistant starch, fructooligosaccharides (FOS), and inulin, have been shown to increase autochthonous bifidobacteria in infants [36–38] and adults [39–44]. However, the relative abundance of resident *Bifidobacterium* levels in adults is highly variable, ranging from 0 to 3% [45–48], and not all subjects respond to prebiotic intervention, even at high doses [45, 46, 49, 50]. Therefore, one approach to enrich for bifidobacteria, increase the number of responders, and enhance their metabolic activity would be to administer a prebiotic together with a select probiotic *Bifidobacterium* strain or strains that use the prebiotic as a growth substrate in vivo. Such pairings are referred to as synergistic synbiotics [51]. According to ecological theory, the provision of resources in a microbial community leads to a relaxation of competition [52, 53] and therefore could enhance colonization success of probiotic strains [52, 53].

Several recent studies have reported improvements in specific health biomarkers or outcomes after consumption of synbiotics [54–56]. Few studies, however, have systematically determined if synbiotics improve the ecological attributes (such as establishment) and/or enhance the health benefits of specific probiotics strains compared to the probiotic alone [57–62]. Moreover, it is currently unknown if it is possible for a probiotic strain to benefit from the presence of a prebiotic substrate in the competitive environment of the human gut. Stable engraftment of an autochthonous *B*. *longum* strain in the human gut was detected in subjects with an apparent open niche based on resources [63], but it is unclear if such substrates can be administered by the diet. It is also possible for prebiotics to exert microbiota-independent effects [64–66].

We have recently developed a method for the selection of autochthonous bacterial strains that are able to benefit from prebiotic substrates in the competitive environment of the gastrointestinal tract [61]. This approach, in vivo selection (IVS), is based on the identification of bacterial strains that became enriched in human fecal samples through the administration of a prebiotic compound, providing a high likelihood that the strain can preferentially utilize the substrate under the exact ecological condition that prevail in the human gut. One such strain, *B*. *adolescentis* IVS-1, was isolated from a human subject, was unique to that subject, and grew well on GOS [45, 61]. Remarkably, when *B*. *adolescentis* IVS-1 was fed to rats, its relative abundance increased from 3% in the absence of GOS to 37% when fed as a synbiotic, i.e., in the presence of GOS [61]. The ability of IVS-1 to expand more than tenfold, even in a different host animal, suggested that this strain could be similarly enriched in human subjects.

The primary goal of this study, therefore, was to systematically compare the ecological and functional properties of a rationally selected synergistic synbiotic in a parallel-arm, placebo-controlled human trial. Treatments included the human autochthonous strain, *B*. *adolescentis* IVS-1, paired with its cognate prebiotic (GOS), as well as a GOS synbiotic containing an allochthonous commercial strain, *B*. *animalis* subsp. *lactis* BB-12 [67]. The latter is known to utilize GOS in vitro and indeed has a higher growth rate on GOS than on glucose [68, 69]. This strain has also been combined with GOS and used previously as a synbiotic in human trials [70–72]. We also included treatments containing only the probiotic strain or the prebiotic. This study design allowed us to assess the ability of the probiotic strains to establish in the gastrointestinal tract, in the presence and absence of GOS, and to identify their effects on microbiota composition. Clinical outcomes were also assessed, with gut permeability as the primary endpoint in a target group susceptible to a leaky gut (obese individuals).

## Methods

### Subjects

This study was a randomized, double-blinded, placebo-controlled, parallel-arm clinical trial conducted at Rush University Medical Center (RUMC) in Chicago, USA. Women and men between 18 and 65 years with a BMI of 30.0–40.0 kg/m^2^ were recruited. Exclusion criteria included the following: (1) prior intestinal resection; (2) patient history of GI diseases except for hiatal hernia, gastroesophageal reflux disease (GERD), and hemorrhoids; (3) severe renal disease defined by creatinine more than twice normal; (4) markedly abnormal liver function defined by ALT/AST over four times normal levels or elevated bilirubin; (5) antibiotic use within the last 12 weeks prior to enrollment; (6) lean or overweight (BMI < 30.0 kg/m^2^), (7) intolerant to aspirin; (8) regular use of aspirin; (9) excessive alcohol intake (more than two drinks for men and one drink for women daily); (10) presence of chronic metabolic disease such as symptomatic cardiovascular disease, insulin-requiring or uncontrolled diabetes, current active treatment of cancer; (11) a plan to have a major change in dietary habit during the following 6 months; (12) consumption of probiotics, prebiotics, or synbiotics without an appropriate 2-week washout period; (13) self-reported lactose intolerance; (14) subjects younger than 18 or older than 65; and (15) unwillingness to consent to the study.

### Study design

**Fig. 1:**
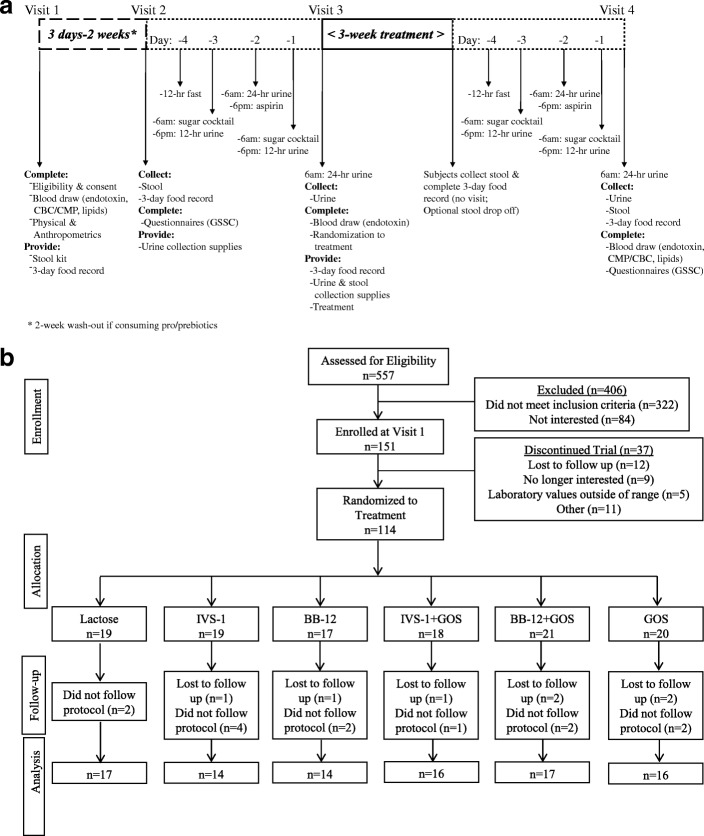
Experimental design and participant flow diagram. **a** Time line for the human trial. Four visits were required from each subject. At visit 1, potential subjects were screened for eligibility and provided with a 3-day food record, all supplies for stool and urine collection (stool kit, urine collection containers, sugar cocktail, and aspirin), and instructions for specimen handling and for completing these tasks before the next visit. Each subject collected stool before taking the sugar cocktail to avoid potential effects of sugar cocktail on microbiota composition. Details of the stool and urine collection are shown. GSSC, Gastrointestinal Symptom and Severity Checklist. **b** Participant flow diagram showing the progress through the phases of the randomized controlled trial (enrollment, intervention allocation, follow-up, and data analysis)

Four visits were required for each subject (Fig. 1a). At visit 1, potential subjects were screened for eligibility and provided written informed consent. Vitals and anthropometrics were completed, and blood was obtained for endotoxin and metabolic markers. Subjects were instructed to collect stool in anaerobic bags before visit 2 and deliver samples within 24 h if stored at − 20 °C or within 5 h if stored at room temperature. At visit 2, study subjects completed a 3-day food record and a standardized 34-item GI symptom questionnaire (GSSC; Gastrointestinal Symptom and Severity Checklist) to identify potential impacts of treatment on GI symptoms, including stool consistency, discomfort, flatulence, abdominal pain, and bloating, on a scale from 0 (best) to 10 (worst). Within 1 week, subjects returned for visit 3 to provide urine for baseline intestinal permeability measurement, completed a blood draw, and were randomized to one of the six treatments (see below). At the end of a 3-week treatment period, subjects returned to the clinic to provide stool and urine samples and to complete a 3-day food record to ensure consistency of dietary intake throughout the study. A blood draw, anthropometrics, and identical follow-up questionnaires were completed. This study was approved by the Institutional Review Board at RUMC in February of 2012, and all procedures were conducted according to the principles expressed in the Declaration of Helsinki. This trial was registered at clinicaltrials.gov, identifier NCT02355210.

### Treatments

The prebiotic, GOS, was obtained from FrieslandCampina, Amersfoort, the Netherlands (sold under the trade name, Vivinal GOS powder) and contained 72.5% GOS, 22.8% lactose, and 4.7% monosaccharides (galactose and glucose). It was previously established that a GOS dose of 5 g per day was sufficient to induce a bifidogenic response [39]. Therefore, the total amount of GOS powder was increased to 6.9 g to achieve a dose of 5 g GOS. An additional 0.1 g of lactose was added to achieve the same weight (7.0 g) as the other preparations.

The two probiotic strains used were *B*. *adolescentis* IVS-1 [61] and *B*. *animalis* subsp. *lactis* BB-12. Strain IVS-1 was produced from a contract manufacturer (Danwell Technology, Garden Grove, CA) and provided as a freeze-dried powder. Strain BB-12 was provided by Chr. Hansen (Hørsholm, Denmark), also as a high cell density freeze-dried powder. Its reported ability to ferment GOS was confirmed in preliminary experiments (data not shown). The probiotic powders were stored for up to 6 months at − 18 °C, and viable cell numbers, as determined by cultural enumeration, were shown to be stable throughout the entire study period (data not shown). Probiotic treatments were portioned into sachets, each containing 0.1 g of probiotic powder (10^10^ CFU/g), resulting in a daily dose of 1 × 10^9^ CFU and 6.9 g of lactose as a carrier/control. Synbiotics contained 6.9 g of Vivinal and 0.1 g of probiotic (either *B*. *adolescentis* IVS-1 or *B*. *animalis* BB-12), for a total dose of 7.0 g. Placebo samples contained 7.0 g of lactose.

The material was packaged in sachets in the Food Processing Product Development Lab at the University of Nebraska. The sachet material was impermeable to oxygen and moisture. Subjects were provided with enough sachets for the entire length of the 3-week treatment period and were instructed to consume one sachet daily, with 2 h between treatment and food consumption in order to facilitate delivery of the probiotic/prebiotic compounds into the cecum/large intestine by rapid emptying of the compounds from the stomach and through small bowel. Subjects were instructed to mix the treatment with the provided bottled water only, as previous testing indicated reduction in bacterial count of IVS-1 with tap water (data not shown). All subjects were instructed to store treatments in their freezer (− 18 °C).

### Analysis of fecal microbiota

Fecal samples were stored in aliquots at − 80 °C until further analysis. The DNA was extracted as previously described [35]. Amplicon sequencing (Illumina MiSeq platform v3 kit producing 300-bp paired-end sequences) was performed at the University of Minnesota Genomics Center, with all samples being included in a single run. The V5-V6 region of the 16S rRNA gene was amplified using primer pair 784F (5′-RGGATTAGATACCC-3′) and 1064R (5′-CGACRRCCATGCANCACCT-3′). These primers were chosen based on our previous findings that the V5-V6 region provided the best resolution among *Bifidobacterium* species [61, 63], which we considered the most important criteria for the aims of our study. The 25-μl PCR mixtures contained 5 μl of template DNA, 5 μl of 2× HotStarTaq PCR master mix, a final concentration of primers of 500 nM, and 0.025 U μl^−1^ HotStarTaq polymerase (Qiagen Inc.), as previously described [61]. The generated sequences were quality filtered with Illumina software at the University of Minnesota Genomics Center, resulting in more than 96% of the samples meeting all quality control criteria. Sequences that did not meet quality filtering criteria were removed from the analysis. All reads were trimmed to 240 base pairs using the FASTX-Toolkit. The reads were merged and analyzed for their sequencing depth. If a sample exceeded 37,000 reads, it was subsampled using Mothur v.1.31.162, while samples that had less than 37,000 sequences were left untreated. This normalization step was done as suggested by Weiss et al. to account for differences in the library size and minimize potential biases due to sequencing depth across samples [73]. Subsequently, reads were filtered by length with a minimum of 240 base pairs and a maximum of 260 base pairs, dereplicated, OTU clustered, chimeras removed, and taxonomically assigned as previously described [61]. After processing and quality control, samples contained an average of 22,487.59 ± 6683 sequences.

### Quantitative real-time PCR (qPCR)

qPCR was performed by using a Mastercycler Realplex2 instrument (Eppendorf AG, Hamburg, Germany). Each PCR was performed with 25-μl volumes using real-time master mix containing SYBR (5 Prime Inc., Gaithersburg, MD) and either genus-specific primers for *Bifidobacterium* [39], or the strain-specific primers for *B*. *adolescentis* IVS-1, as described previously [61]. Strain-specific PCR for IVS-1 did not reveal products in baseline samples in subjects of groups IVS-1 or IVS-1 + GOS or in the baseline of another additional 20 randomly selected subjects (data not shown).

For strain-specific detection of *B*. *animalis* subsp. *lactis* BB-12, the PCR mixture contained 25 μl of PCR reaction mix (QuantiFast® Probe PCR Kit, QIAGEN, Hilden, Germany), 0.3 μmol of each primer (BAL-23S-F 5′-CAGGTGGTCTGGTAGAGTATACCG-3′ and BAL-23S-R 5′-ACGGCGACTTGCGTCTTG-3′), 0.25 μmol of probe (BAL-23S-P 5′-FAM-CGCCCACGACCCGCAAG-TAMRA-3′), and 5 μl DNA as previously described [74]. The target of these primers and probe is the elongation factor Tu (tuf) gene of BB-12. The specificity of the primers and probe for BB-12 was validated experimentally by qPCR using DNA from 11 different *Bifidobacterium* strains using the same approach as described previously for the IVS-1 strain-specific qPCR [61]. These strains included *B*. *adolescentis* IVS-1, *B*. *adolescentis* ATCC 15703, *B*. *adolescentis* L2-32, *B*. *longum* subsp. *longum* ATCC 15707, *B*. *longum* DJO10A, *B*. *longum* ATCC 15697, *B*. *longum* subsp. *longum* F8, *B*. *longum* subsp. *longum* JDM301, *Bifidobacterium* sp. strain 113, *Bifidobacterium* sp. strain 12_1_47BFAA, and *Bifidobacterium* sp. strain HMLN14. Furthermore, primers were tested against fecal DNA obtained from baseline fecal samples from subjects in BB-12 and BB-12 + GOS groups, and additional randomly selected samples from other subjects. Strain BB-12 was detected in three out of 51 tested subjects before the treatment was started, which may be due to accidental consumption of food products containing this strain.

Overall, we concluded that both qPCR assays were sufficiently specific and that the test strains were either absent or rare in the pre-treatment microbiota of the subjects. Absolute quantification of both strains was achieved through standard curves prepared by tenfold dilutions of DNA isolated from overnight cultures (14 h) for which cell numbers were determined by quantitative culture.

### Intestinal permeability

Subjects ingested a sugar mixture containing 2 g mannitol, 7.5 g lactulose, 40 mg sucrose, and 2 g sucralose after a 12-h overnight fast (Fig. 1a). Subjects collected urine into three separate containers for 5, 7, and 12 h, for a total collection time of 24 h, in order to estimate gastroduodenal permeability (5 h urinary sucrose), proximal and distal small bowel permeability (5 h and first 12 h urinary mannitol and lactulose and lactulose:mannitol ratio), and total and primarily colonic permeability (24 h urinary sucralose level and sucralose/lactulose ratio) as we previously described [75]. Compared to normal-weight individuals, obese individuals are more likely to have a hyper-permeable intestine [76]. However, values vary greatly among individuals, and a treatment with aspirin has been shown to improve consistency in intestinal permeability measurements [76, 77]. Therefore, the subjects participated in an aspirin challenge consuming four 325-mg aspirin tablets both 12 h before and immediately before ingestion of the sugar mixture. Thus, urine was collected for two separate 24-h periods with and without aspirin treatment, and both before and after treatment. Urine was analyzed for concentrations of mannitol, lactulose, and sucralose using gas chromatography (GC). Intestinal permeability was expressed as a percent of the oral dose excreted in the urine.

### Plasma and serum measures of endotoxin exposure

Serum endotoxin (LPS) and LPS-binding protein (LBP) were measured after the aspirin challenge both before and after treatment. Endotoxin was measured in serum by Limulus Amebocyte Lysate QCL-1000 (Lonza # 50-647U). Serum samples were diluted at a 1:5 ratio with LAL reagent water. LBP was measured in plasma using an ELISA kit from Cell Sciences Inc. (# HK315).

### Serum metabolic markers

A complete metabolic panel and complete blood count were performed to allow an assessment of treatment safety. To assess impact of treatments on metabolic markers, a lipid panel was completed by Quest Diagnostics and included the following: total cholesterol (TC), low-density lipoprotein cholesterol (LDL-C), high-density lipoprotein cholesterol (HDL-C), and triglycerides. Non-HDL was calculated by subtracting HDL-C from TC.

### Vitals and anthropometrics

Blood pressure was measured using an automated cuff with the average of three assessments used for statistical comparisons. Body weight and waist circumference was assessed and BMI calculated before and after treatment.

### Statistical analysis

All data presented was analyzed based on a per protocol analysis. Subjects were excluded from the analysis if the study protocol was not followed, including use of antibiotics during the treatment period, storage of the treatments at room temperature, and use of tap water to consume treatments. Data is presented as mean ± SEM for variables that were normally distributed, or median (IQR) for variables not normally distributed. Group means were compared by ANOVA and post hoc tests except when data were not normally distributed, in which case a nonparametric analysis of medians was performed using the Kruskal-Wallis test. Chi-square tests or Fisher’s exact tests were used for incidence data. If only two groups were compared, Student’s *t* tests were performed.

For the analysis of the gut microbiota, Mann-Whitney-Wilcoxon matched pair tests were used for pairwise comparisons between time points for the 16S rRNA gene sequencing and qPCR data, as data was normally distributed. Unpaired Mann-Whitney-Wilcoxon tests were used for pairwise comparisons between treatment groups. When more than two samples were compared, Kruskal-Wallis test was applied. *P* values were corrected for the total number of comparisons with the use of a false-discovery rate (FDR) method in R, whereby values were considered significant for FDR-adjusted *P* values (reported as *Q* values) of < 0.1.

## Results

### Subject enrollment

A total of 151 volunteers were enrolled in the study (Fig. 1b). Of these, 114 subjects were randomly assigned to the six treatments, and 94 were used in the analyses after accounting for attrition and excluding for protocol deviation.

### Baseline demographic and clinical characteristics

The majority of the subject cohort was female (71%), middle aged (44.3 ± 11.2 [mean ± SD]), non-Hispanic or Latino (90.4%), and of African American ethnicity (61.7%) (Additional file 1: Table S1). All subjects were obese, with a median (IQR) BMI of 36.7 (8.5) kg/m^2^ and waist circumference of 45.0 ± 7.3 in. Other clinical metabolic markers were within the normal range (Additional file 1: Table S1). Randomization resulted in all demographic and clinical characteristics, including race, ethnicity, systolic/diastolic blood pressure, cholesterol (total, LDL, HDL, non-HDL), glucose, and triglyceride, to not differ significantly between groups with one exception: the IVS-1 + GOS group had a higher BMI (*P* = 0.049) when compared to the IVS-1 group (Additional file 1: Table S1).

### Safety and tolerability of treatments and impact on dietary patterns

No differences in complete metabolic panel and complete blood count values were observed with any treatment (data not shown). Mild gastrointestinal symptoms are common among heathy obese adults, and many study participants reported bloating (60.6%), passing gas (85.1%), hard stools (46.8%), and watery stools (43.6%) at baseline. No significant differences in median symptom score were detected between the six groups at baseline (Additional file 1: Table S2). Treatments were generally well tolerated with minimal reported side effects. The GOS group had significantly harder stools when compared to the BB-12 + GOS group (*P* = 0.024). Passing gas increased from a median of 2.5 to 5.0 with lactose supplementation, potentially because of undeclared lactose intolerance, but this was not significantly different between baseline and treatment end (*P* = 0.150). The “severity of passing gas” was significantly reduced from 4.0 to 1.0 in the BB-12 + GOS group (comparison of baseline to treatment; *P* = 0.040), and severity of hard stools increased from 1.0 to 3.5 (on a score from 1 to 10) in the GOS group (*P* = 0.030). No differences in macronutrient or micronutrient intake were detected before and after the treatment period, suggesting treatment tolerability was not influenced by dietary intake (data not shown).

### Cell numbers of strains IVS-1 or BB-12 in fecal samples

**Fig. 2:**
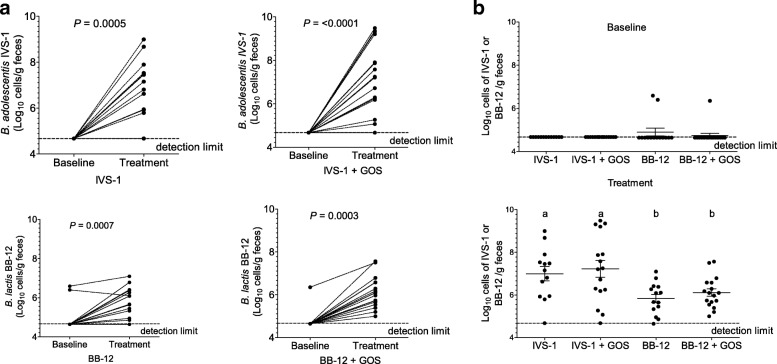
Quantification of probiotic strains in fecal samples by qPCR. **a** Quantification of absolute cell numbers of probiotic strains in fecal samples by strain-specific qPCR. Shown are probiotic and synbiotic treatment groups at baseline and treatment time points. Significance of *P* ≤ 0.05 is denoted by a single asterisk (*), *P* ≤ 0.01 by two asterisks (**), and *P* ≤ 0.001 by three asterisks (***). **b** Direct comparison of absolute abundances of *B*. *adolescentis* IVS-1 and *B*. *animalis* subsp. *lactis* BB-12 at each time point using strain-specific qPCR. Different letters indicate significant differences between groups (*P* ≤ 0.05)

Strain-specific qPCR revealed that both strains reached significantly higher cell numbers during the treatment period when compared to the baseline samples (*P* < 0.001 in group IVS-1 + GOS; *P* < 0.0007 in groups IVS-1, BB-12, and BB-12 + GOS) (Fig. 2a). IVS-1 was detectable at an average of 6.99 ± 1.2 log_10_ and 7.22 ± 1.6 log_10_ of cells g^−1^ in the IVS-1 and IVS-1 + GOS groups, respectively. BB-12 was detected at absolute numbers of 5.83 ± 0.7 log_10_ and 6.11 ± 0.7 log_10_ cells g^−1^ in the BB-12 and BB-12 + GOS groups, respectively. Comparisons of cell numbers of the probiotic strains among treatments (Fig. 2b) revealed that IVS-1 was detected at significantly higher numbers in fecal samples than BB-12 in both probiotic-only treatments (*P* = 0.0056), and when GOS was added (*P* = 0.0127). Although GOS led to a modest increase in cell numbers of both strains when compared with the probiotic-only groups (Fig. 2b), this increase did not reach statistical significance (*P* = 0.6682 and *P* = 0.3034, respectively).

### Impact of treatments on total number of bifidobacteria in fecal samples

**Fig. 3:**
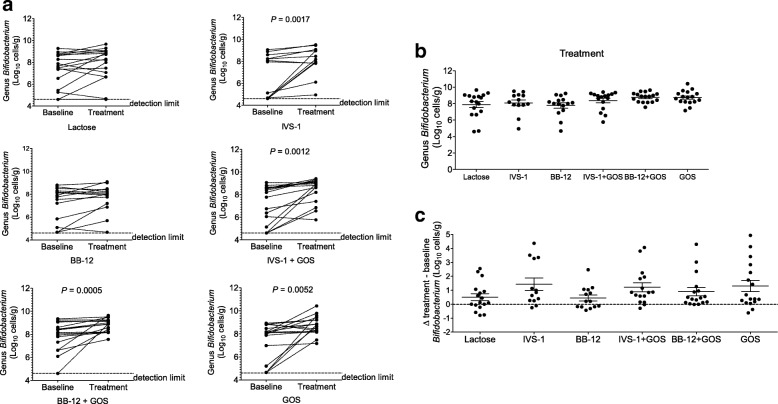
Absolute quantification of total bifidobacteria by qPCR. **a** Quantification of absolute cell numbers of total bifidobacteria in fecal samples by genus-specific qPCR. **b** Direct comparison of abundances of genus *Bifidobacterium* at the treatment time point using genus-specific qPCR. **c** Change in abundance of bifidobacteria for each subject with treatment consumption

Genus-specific qPCR was used to determine total fecal bifidobacteria in all six groups (Fig. 3a). There was no significant difference in the numbers of *Bifidobacterium* in the baseline samples between groups. Among the probiotic treatments, only IVS-1 administration led to an increase in the total number of bifidobacteria (*P* = 0.0017). Significant increases were also detected for all three groups containing GOS when compared to baseline, confirming the bifidogenic effect of GOS that has been observed in previous human studies [40, 41, 45, 78–80].

There was no significant difference in the average numbers of *Bifidobacterium* between treatment groups at the end of the treatment period (Fig. 3b). However, the *Bifidobacterium* numbers at the baseline varied greatly between individuals within each group, ranging from the detection limit (log_10_ 4.7 cells g^−1^ feces) to a maximum of 10.4 log_10_ cells g^−1^ feces. In addition, a large variation in the increase in total bifidobacteria was detected among participants (Fig. 3a), suggesting an individualized response to the treatments in all six groups. Therefore, the absolute changes in *Bifidobacterium* cell numbers for individual subjects were calculated and these shifts compared among the six groups (Fig. 3c). Although there were no significant differences (*P* = 0.2071), the highest increases in *Bifidobacterium* numbers were found in the IVS-1 group with 1.43 ± 1.6 log_10_, followed by the GOS group with 1.30 ± 1.7 log_10_. Interestingly, the lactose group also showed a modest increase in bifidobacteria (0.50 ± 1.0 log_10_).

### Characterization of the fecal microbiota by 16S rRNA gene sequencing

Illumina sequencing of 16S rRNA gene amplicons was used to determine the effect of the treatments on the overall bacterial community in fecal samples. This analysis revealed that the treatments did not exert a community-wide effect on the resident gut microbial community: Both alpha- and beta-diversity were compared between time points within each group, and across all six groups for both time points, but no differences were detected for any of the comparisons (*P* > 0.05, data not shown).

Illumina sequencing, however, did show that the relative abundances of specific taxa were affected by treatments. In particular, the phylum, Actinobacteria, were significantly higher in subjects treated with IVS-1 (*P* = 0.0072, *Q* = 0.0316), IVS-1 + GOS (*P* = 0.0015, *Q* = 0.0176), BB-12 + GOS (*P* = 0.0279, *Q* = 0.0516), GOS (*P* = 0.0468, *Q* = 0.0722), and lactose (*P* = 0.0526, *Q* = 0.0794) (Table 1). At the genus level, the relative abundance of *Bifidobacterium* was increased in groups IVS-1 (*P* = 0.0138, *Q* = 0.0420), IVS-1 + GOS (*P* = 0.0039, *Q* = 0.0253), BB-12 + GOS (*P* = 0.0140, *Q* = 0.0420), and GOS (*P* = 0.0686, *Q* = 0.0991).

**Table 1:** Proportions of bacterial taxa (with > 0.1% in at least one of the treatments) significantly influenced by dietary treatments within treatment groups (FDR-adjusted *P* value < 0.1). Significant values are set in italics

Treatment					
	Taxonomic group	Baseline	Treatment	*P* value	*Q* value (FDR adjusted)
IVS-1	Phylum				
	Actinobacteria	9.106 ± 5.25	15.422 ± 5.98	*0.0072*	*0.0316*
	Genus				
	*Bifidobacterium*	7.605 ± 7.41	14.565 ± 6.92	*0.0138*	*0.0420*
	OTUs^a^				
	OTU_1 (*B*. *adolescentis*)	1.199 ± 4.24	3.403 ± 5.08*	*0.0010*	*0.0176*
	OTU_167 (*B*. *animalis* subsp. *lactis*)	0.001 ± 0.00	0.001 ± 0.00	1.0000	1.0000
	OTU_7 (*B*. *adolescentis or ruminantium*)	0.817 ± 2.24	1.916 ± 4.33	*0.0291*	*0.0516*
Bb 12	Phylum				
	Actinobacteria	10.660 ± 5.17	11.886 ± 7.68	0.9051	0.9540
	Genus				
	*Bifidobacterium*	8.796 ± 5.70	9.450 ± 8.39	0.8782	0.9514
	OTUs^a^				
	OTU_1 (*B*. *adolescentis*)	1.237 ± 2.46	1.160 ± 1.62	0.7787	0.8814
	OTU_167 (*B*. *animalis* subsp. *lactis*)	0.034 ± 0.04	0.055 ± 0.05	*0.0073*	*0.0316*
IVS-1 GOS	Phylum				
	Actinobacteria	8.129 ± 4.79	15.780 ± 7.11	*0.0015*	*0.0176*
	Genus				
	*Bifidobacterium*	5.959 ± 5.88	14.650 ± 7.44	*0.0039*	*0.0253*
	*Anearotrunctus*	0.048 ± 0.09	0.010 ± 0.01	*0.0236*	*0.0511*
	*Roseburia*	3.379 ± 1.64	1.825 ± 1.15	*0.0018*	*0.0176*
	OTUs^a^				
	OTU_1 (*B*. *adolescentis*)	2.476 ± 4.49	7.344 ± 7.00*	*0.0176*	*0.0476*
	OTU_167 (*B*. *animalis* subsp. *lactis*)	0.001 ± 0.00	0.003 ± 0.01	1.0000	1.0000
	OTU_102 (*Lachnospiracea incertae sedis*)	0.352 ± 0.49	0.070 ± 0.16	*0.0100*	*0.0390*
	OTU_152 (*Alistipes*)	0.176 ± 0.36	0.013 ± 0.05	*0.0463*	*0.0722*
Bb 12 GOS	Phylum				
	Actinobacteria	11.665 ± 5.90	17.034 ± 6.51	*0.0279*	*0.0516*
	Genus				
	*Bifidobacterium*	8.862 ± 6.15	15.86 ± 7.97	*0.0140*	*0.0420*
	*Anaerovorax*	0.295 ± 0.09	0.18 ± 0.09	*0.0283*	*0.0516*
	OTUs^a^				
	OTU_1 (*B*. *adolescentis*)	3.138 ± 4.53	6.797 ± 9.61	0.0812	0.1131
	OTU_167 (*B*. *animalis* subsp. *lactis*)	0.003 ± 0.001	0.112 ± 0.18	*0.0006*	*0.0176*
	OTU_156 (*Clostridium* XI)	0.151 ± 0.20	0.071 ± 0.21	*0.0345*	*0.0585*
GOS	Phylum				
	Actinobacteria	13.923 ± 8.98	18.067 ± 9.78	*0.0468*	*0.0722*
	Genus				
	*Bifidobacterium*	11.329 ± 10.48	17.358 ± 11.41	*0.0686*	*0.0991*
	*Bacteroides*	5.794 ± 4.43	2.854 ± 4.11	*0.0121*	*0.0420*
	OTUs^a^				
	OTU_1 (*B*. *adolescentis*)	3.793 ± 7.47	6.645 ± 8.83	0.1221	0.1587
	OTU_167 (*B*. *animalis* subsp. *lactis*)	0.000 ± 0.00	0.203 ± 0.85	0.4504	0.5489
Lactose	Phylum				
	Actinobacteria	10.471 ± 6.75	14.502 ± 6.89	*0.0529*	*0.0794*
	Genus				
	*Bifidobacterium*	7.895 ± 8.10	12.008 ± 8.02	0.0917	0.1233
	OTUs^a^				
	OTU_1 (*B*. *adolescentis*)	1.241 ± 1.93	2.557 ± 4.11	0.6562	0.7755
	OTU_167 (*B*. *animalis* subsp. *lactis*)	0.000 ± 0.00	0.037 ± 0.14	0.7910	0.8814
	OTU_315 (*Coprobacillus*)	0.100 ± 0.09	0.062 ± 0.06	*0.0023*	*0.0179*
	OTU_43 (*Ruminococcus*2)	0.562 ± 0.75	0.272 ± 0.63	*0.0051*	*0.0284*
	OTU_180 (*Bacteroides*)	0.041 ± 0.17	0.127 ± 0.40	*0.0183*	*0.0476*

^a^If the strain could not be assigned to a type strain (< 97% homology), RDP Classifier was used to determine the most likely genus (80% cutoff)

*Significant difference between the two treatment groups for OTU_1

The most significant and consistent shifts were detected among the operational taxonomic units (OTUs) that represented the probiotic strains. The OTU with an identical sequence to *B*. *adolescentis* IVS-1 (OTU_1) was significantly enriched in groups IVS-1 (*P* = 0.0010, *Q* = 0.0176) and IVS-1 + GOS (*P* = 0.0176, *Q* = 0.0476), indicating that this OTU was primarily enriched through the administration of the probiotic (although non-significant increases were also detected for the GOS and lactose groups). Mann-Whitney test between groups IVS-1 and IVS-1 + GOS showed that IVS-1 + GOS had a significantly higher relative abundance of OTU_1 than IVS-1 (*P* = 0.0146), suggesting a functional synergism between the synbiotic components IVS-1 and GOS. Similar synergism was not detected for OTU_167 representing *B*. *animalis* subsp. *lactis* BB-12 in the BB-12 and GOS groups (*P* = 0.6895). However, this OTU was still significantly increased in both groups when compared to baseline. It was also mostly undetectable in groups that did not receive BB-12 (confirming its allochthonous status in the human gut).

Interestingly, the enrichment of OTU_1 with GOS showed associations with other members of the *Bifidobacterium* population. An analysis of individuals that had no, or only a low increase of OTU_1 (calculated as relative abundance of OTU_1 of total *Bifidobacterium* rather than of total bacteria) in groups IVS-1 and IVS-1 + GOS at baseline (non-responders), showed that these subjects had one of six other *Bifidobacterium* OTUs to be dominant at baseline. The six identified OTUs included OTU_7 (*B*. *adolescentis* or *ruminantium*), OTU_10 (*B*. *longum*), OTU_2055 (*B*. *pseudocatenulatum*), OTU_2111 (*Bifidobacterium* sp.), OTU_2202 (*B*. *pseudocatenulatum*), and OTU_438 (*B*. *pseudocatenulatum*). These OTUs are related to OTU_1 (Additional file 2: Figure S1). None of these six OTUs correlated significantly with OTU_1 when analyzed on their own. However, the sum of the six OTUs correlated negatively with the relative abundance of OTU_1 at the end of the treatment period (*P* = 0.0313, *R* = − 0.4005), indicating competition between these taxa and OTU_1 for the substrate GOS.

Few other changes were detected. We tested an effect of the treatments on specific features of the microbiota that have been suggested to be relevant for health. The ratio between *Prevotella* and *Bacteroides*, which has been reported to be influenced by dietary treatments and long-term dietary patterns [81, 82] and relevant for metabolic health [81, 83], was not different within treatment groups or when groups were compared (data not shown). Additionally, the relative abundance of butyrate producing genera such as *Faecalibacterium*, *Eubacterium*, *Roseburia*, *Lachnobacterium*, and *Ruminococcus* was analyzed. IVS-1 + GOS supplementation significantly decreased the relative abundance of *Roseburia* (Table 1). We also analyzed the shift in relative abundances (treatment value minus baseline value) and saw a significant reduction of *Lachnobacterium* (*P* < 0.0001) in the GOS group (data not shown).

### Impact of treatments on intestinal permeability and endotoxemia

**Fig. 4:**
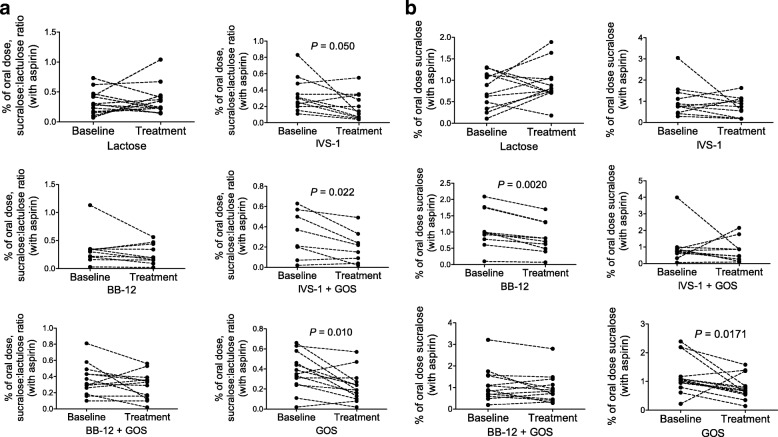
Intestinal permeability after an asperin challenge as inferred by measuring the **a** sucralose:lactulose ratio and **b** concentration (as percent of oral dose) in urine before and after the pro-, pre-, and synbiotic treatments. To assess intestinal permeability, urine was collected for 24 h after the subjects had consumed a sugar cocktail together with aspirin and analyzed by gas chromatography

To determine the effect of the treatments on intestinal permeability, urine sugar excretion after consumption of a sugar cocktail was compared in pre-treatment samples versus samples taken after the interventions, and LPS and LBP levels were determined in serum. At baseline, no significant differences in gastrointestinal permeability existed between the groups (*P* > 0.4, data not shown). No differences in percent change in intestinal permeability were detected between the groups after treatment (inter-group comparisons; Additional file 1: Table S3). When differences in absolute permeability were examined before and after treatment by intra-group comparisons, there were significant reductions in permeability for the post-aspirin sucralose:lactulose ratio in groups IVS-1 (*P* = 0.050), IVS-1 + GOS (*P* = 0.022), and GOS (*P* = 0.010) (Fig. 4a). Additionally, there was a significant reduction in the post-aspirin excretion of sucralose in groups BB-12 (*P* = 0.0020) and GOS (*P* = 0.0171) (Fig. 4b). Permeability in the absence of an aspirin challenge was not affected (data not shown).

Baseline markers of endotoxemia did not differ between groups (*P* > 0.05). There were no significant effects of any treatment on serum LPS and LBP levels (*P* > 0.05, Additional file 1: Table S4). Minimal changes were seen in both anthropometrics and metabolic markers; the only significant difference was seen between a median increase in HDL-cholesterol in the BB-12 + GOS (3.7 [15.9]) group and a decrease in the BB-12 group (− 3.8 [13.2]) (*P* = 0.032, Additional file 1: Table S5).

## Discussion

In this study, we characterized both the ecological impact and physiological effect of two synbiotic combinations, each composed of a *Bifidobacterium* strain and the prebiotic GOS, with each other and with their probiotic and prebiotic components. Despite the rational for the synbiotic approach, establishing synergism (ecological or functional) has been challenging for several important reasons [62]. First, most probiotic/prebiotic combinations have been designed and formulated without having demonstrated synergism under the competitive conditions of the gastrointestinal tract. Second, rarely has the experimental design of studies included individual probiotic and synbiotic treatments. Finally, most synbiotic studies used techniques that did not allow for a quantification of the probiotic strain. In this study, we compared synbiotic products comprised of either an in vivo selected autochthonous or an allochthonous strain, each paired with GOS and tested by itself, and included a qPCR analysis with strain level resolution to quantify the probiotic strains.

Consistent with ecological theory [53], the autochthonous strain IVS-1 had a clear ecological advantage over the allochthonous strain BB-12 in that it could be established at about tenfold higher cell numbers. In addition, administration of IVS-1 increased cell numbers of total bifidobacteria as well as the proportion of the genus *Bifidobacterium* and the phylum Actinobacterium. Such enrichments of higher taxonomic groups through the administration of probiotic bifidobacteria has, to our knowledge, not been previously shown and is remarkable given that the human gut contains high level of resident bifidobacteria and Actinobacteria. In contrast, the allochthonous strain BB-12, although detectable at low levels with 16S rRNA gene sequencing, did not increase total bifidobacteria. Overall, the findings support the concept that autochthonous members of the microbiota would be substantially more successful when introduced into the human gut [53, 63].

The findings on the ability of GOS to support the probiotic strains were more subtle. The OTUs that represented the two probiotic strains (OTU_1 and OTU_167) were higher in the synbiotic groups when compared to the probiotic groups alone, with findings reaching significance for OTU_1 (Table 1). However, cell numbers of both IVS-1 and BB-12 were only marginally and not significantly increased through GOS (Fig. 2). We therefore conclude that although there was a consistent increase in the probiotic strains in the synbiotic groups, inter-individual variability was likely too high to achieve significant findings with the number of subjects in this study. Our findings further suggest that ecological interactions with the resident microbiota but also the specific manner in which the synbiotic preparations were formulated (e.g., all included lactose, and GOS doses were all at 5 g) could explain the limited synergism that we observed.

The analysis of the whole gut bacterial community by 16S rRNA gene sequencing not only provided insights into the effect of the treatments on overall microbiota composition, but also suggested a possible reason for the limited synbiotic effect that we observed. Overall, the effect of the treatments on gut microbiota composition was remarkably specific, increasing mainly total bifidobacteria and the specific OTUs that represented the probiotic strains, while a limited number of other taxa were decreased (Table 1). In agreement with our previous study [45], the most significant increase induced by GOS was in the genus *Bifidobacterium*, which occurred in all groups receiving GOS, including the groups that did not receive the probiotic strains. This finding clearly establishes that resident members of the gut microbiota are able to utilize GOS, likely competing with the ingested probiotic strains for the substrate. Accordingly, the OTU representing IVS-1 (OTU_1) correlated negatively with the sum of dominant *Bifidobacterium* OTUs during treatment with GOS, suggesting competition for the substrate. Given that the gut microbiota is highly specific to an individual, it is likely that resident strains have a higher affinity to GOS compared to the incoming strain even if the latter is autochthonous to humans, as it is still foreign to the microbiota of that specific subject.

Another reason for the limited synergistic effect of GOS might be that the *Bifidobacterium* strains in the “probiotic only” powders were given as a mixture with lactose, which also served as the placebo in this study. The rational for that choice was that the GOS is synthesized from lactose and contained 22.8% lactose in the powder that was administered. We further reasoned that lactose would be hydrolyzed and absorbed in lactose-tolerant individuals before it reaches the colon [84]. However, in subjects with insufficient expression of the *LCT* gene (encoding for lactase) [85, 86], lactose can become a “conditional prebiotic” [87] and serve as a colonic substrate for bifidobacteria, which are well equipped to metabolize lactose [88, 89]. While our study subjects self-reported to be lactose tolerant, both the qPCR and amplicon sequencing analyses showed that lactose supplementation did lead to a non-significant and highly personalized increase in the average relative abundance of bifidobacteria. Indeed, seven of the 17 subjects in the lactose group had increases of bifidobacteria of more than 0.50 log_10_ (Fig. 3c). Interestingly, six of these seven “responders” self-identified as African American, who are more likely to carry *LCT* genes that predispose them to lactose malabsorption [85, 86]. A mild form of lactose malabsorption was indeed suggested among our subjects by the non-significant increase in passing gas in the lactose group (Additional file 1: Table S5). Therefore, a combination of the “conditional prebiotic” effect of lactose, the ability of the resident microbiota to compete for GOS, and the substantial inter-individual variation might have lowered our ability to detect a more pronounced impact of GOS on establishment of the probiotic strains.

We chose intestinal barrier function as the primary outcome of the human trial because of its importance in the prevention of inflammation not only in metabolic diseases but also in gastrointestinal and systemic inflammatory disorders such as inflammatory bowel disease [90], non-alcoholic steatohepatitis (NASH) [91], and Parkinson disease [92]. In our study, no treatments significantly impacted markers of intestinal permeability in non-aspirin treated subjects. However, when susceptibility to intestinal hyperpermeability was increased through a high-dose of aspirin, permeability was reduced with both probiotic treatments and with GOS intake. While no overall changes in the microbial community were seen, as measured by alpha- and beta-diversity, the enrichment in bifidobacteria common to all of these treatments might be associated with this decrease, and both the autochthonous and the allochthonous probiotic strain showed an effect. As markers of small bowel permeability (5-h urinary lactulose or mannitol; data not shown) were not reduced, the treatment effects appear to be primarily directed to permeability of the colon [75], the site where the majority of bacteria, including bifidobacteria, reside. Overall, our findings are in agreement with observations in rodents that showed that GOS [93], *B*. *adolescentis* [32], and *B*. *animalis* subsp. *lactis* CNCM-12494 [94, 95] improved intestinal permeability. In accordance with the findings from the gut microbiota analysis, there was no evidence for synergism in the synbiotic treatments in respect to permeability. Indeed, although permeability (based on sucralose excretion) was improved for both the BB-12 and GOS treatments, the BB-12 + GOS treatment was not improved. It is possible that the effect was muted when both were present, or that an antagonistic event had occurred, similar to that recently described by Schroeder et al. [96], where mucus permeability was improved by inulin, but not by inulin plus *B*. *longum*.

Despite the detectable improvement in intestinal permeability, no significant effects on serum endotoxin or metabolic outcomes were seen with any synbiotic, prebiotics, or probiotic treatment. While promising, limited evidence exists to support the use of GOS [97] and bifidobacteria [98] supplementation for serum LPS reduction. In contrast, a synbiotic comprised of *Lactobacillus plantarum* and FOS was recently shown to significantly reduce sepsis in infants [55]. In other studies, improvements in metabolic markers with GOS intake [41] have been observed, but the impact on metabolic outcomes with bifidobacteria intake is uncertain. Bifidobacteria cell counts were increased through IVS-1 + GOS; however, changes in bifidobacteria are not always associated with improvements in LPS [99] or metabolic markers [100]. It is possible that the treatment duration of 3 weeks was not long enough for metabolic improvements to become evident, or that serum concentrations of these markers were not at high enough concentrations to see a meaningful decrease with treatment. Future clinical trials are warranted, targeting pathologies with an underlying leakiness of the gut, using disease-specific endpoints and longer durations of the treatments. Given that we did not observe synergism between GOS and the probiotic strains, future trials should probably focus on single components, or explore higher doses of GOS in synbiotic combinations.

## Conclusion

This study provided an important test of the synbiotic concept. The findings showed that autochthony of a bacterial strain is more important than the provision of prebiotic substrate at a 5-g dose for the establishment of a probiotic in the human gut. This outcome is likely due to the highly competitive environment that favors autochthonous strains that possess traits that allow colonization while effectively competing for substrates [53]. In addition, although both the probiotic strains and the prebiotic GOS improved barrier function, a combination of the two did not result in apparent synergism. It is unclear how competition for the prebiotic can be avoided, but it is possible that higher doses may be necessary. We selected the daily amount of GOS in this study based on the dose that has previously been shown to induce a bifidogenic effect [39], which was confirmed in this study, and successfully enrich the strain IVS-1 in humans [61]. However, for synbiotic approaches, it might be necessary to give doses of prebiotics that exceed those sufficient for bifidogenic effects to ensure that substrates are available for both the resident microbiota and the incoming microbe. Accordingly, in our previous study in rats, a GOS dose that was approximately 30-fold higher when adjusted by bodyweight did result in clear synergism and increased competiveness of IVS-1 [61]. Although the synbiotic approach tested in this study did not provide measurable synergism, our findings clearly show that both probiotic strains and the prebiotic improved markers of intestinal permeability. Thus, this report provides a basis for the use of these treatments (or combinations thereof) in pathologies with an underlying leakiness of the gut.

## Additional files

Additional file 1: **Table S1.** Baseline demographic and metabolic characteristics of study subjects by treatment group. **Table S2.** Differences in gastrointestinal symptoms by treatment group. **Table S3.** Percent change in intestinal permeability in subjects by treatment group. **Table S4.** Differences in markers of endotoxemia by treatment group. **Table S5.** Percent change in anthropometrics and metabolic markers in subjects by treatment group. (DOCX 49 kb)

Additional file 2: **Figure S1.** Phylogenetic analysis of OTU_1 and six closely related *Bifidobacterium* OTUs that might have competed with OTU_1 for the niche in the GI tract using Maximum Likelihood method. OTU_2281 *Lactobacillus animalis* was selected as the Outgroup. Numbers indicate branch lengths. (PPTX 41 kb)

## Abbreviations

ALT: Alanine aminotransferase
AST: Aspartate aminotransferase
BMI: Body mass index
CFU: Colony-forming units
GI: Gastrointestinal
GOS: Galactooligosaccharide
HDL-C: High-density lipoprotein cholesterol
LAL: Limulus amebocyte lysate
LBP: LPS-binding protein
LDL-C: Low-density lipoprotein cholesterol
LPS: Lipopolysaccharide
OTU: Operational taxonomic unit
TC: Total cholesterol
