# Supplementary material for: Probiotic Bifidobacterium strains and galactooligosaccharides improve intestinal barrier function in obese adults but show no synergism when used together as synbiotics
Source: Microbiome. 2018 Jun 28;6:121. doi: 10.1186/s40168-018-0494-4 (PMC6022452; doi:10.1186/s40168-018-0494-4)
Supplement: Supplementary file 1 — Table S1. Baseline demographic and metabolic characteristics of study subjects by treatment group. Table S2. Differences in gastrointestinal symptoms by treatment group. Table S3. Percent change in intestinal permeability in subjects by treatment group. Table S4. Differences in markers of endotoxemia by treatment group. Table S5. Percent change in anthropometrics and metabolic markers in subjects by treatment group. (DOCX 49 kb) [file 40168_2018_494_MOESM1_ESM.docx]

**Additional file 2**

| Table S1: Baseline demographic and metabolic characteristics of study subjects by treatment group^1^ | | | | | | | |
| --- | --- | --- | --- | --- | --- | --- | --- |
|  | **Total Sample (n=94)** | **Control (n=17)** | **IVS-1 (n=14)** | **Bb12 (n=14)** | **IVS-1+GOS (n=16)** | **Bb12+GOS (n=17)** | **GOS**  **(n=16)** |
| **Demographic Characteristics** |  |  |  |  |  |  |  |
| Gender |  |  |  |  |  |  |  |
| Female, n (%) | 71 (75.5) | 13 (76.5) | 9 (64.3) | 12 (75.0) | 11 (78.6) | 12 (75.0) | 12 (75.0) |
| Age, years, mean ± SD | 44.3±11.2 | 43.9 ± 8.8 | 44.7 ± 13.3 | 43.9 ± 12.5 | 44.2 ± 11.8 | 43.1 ± 12.7 | 45.9 ± 9.6 |
| Race, n (%) |  |  |  |  |  |  |  |
| Hispanic/  Latino | 9 (9.6) | 0 (0.0) | 2 (14.3) | 2 (14.3) | 2 (12.5) | 2 (11.8) | 1 (6.3) |
| Non-Hispanic/  Latino | 85 (90.4) | 17 (100.0) | 12 (85.7) | 12 (85.7) | 14 (87.5) | 15 (88.2) | 15 (93.8) |
| Ethnicity, n (%) |  |  |  |  |  |  |  |
| White | 31 (33.0) | 5 (29.4) | 8 (57.1) | 4 (28.6) | 3 (18.8) | 6 (35.3) | 5 (31.3) |
| African  American | 58 (61.7) | 10 (58.8) | 6 (42.9) | 7 (50.0) | 13 (81.3) | 11 (64.7) | 11 (68.8) |
| Other**^2^** | 5 (5.3) | 2 (11.8) | 0 (0.0) | 3 (21.4) | 0 (0.0) | 0 (0.0) | 0 (0.0) |
| Education, n (%) |  |  |  |  |  |  |  |
| ≤ 12 years | 20 (21.3) | 3 (17.6) | 1 (7.1) | 2 (14.3) | 6 (37.5) | 2 (11.8) | 6 (37.5) |
| > 12 years | 74 (78.7) | 14 (82.4) | 13 (92.9) | 12 (85.7) | 10 (62.5) | 15 (88.2) | 10 (62.5) |
| **Clinical Characteristics^3^** |  |  |  |  |  |  |  |
| Body weight, kg**^4^** | 100.0 (25.7) | 96.8 (17.7) | 94.8 (14.6) | 98.5 (32.2) | 118.0 (36.7) | 112.8 (31.8) | 102.3 (18.3) |
| BMI, kg/m^2^ **^5^** | 36.7 (8.5) | 34.0 (4.5) | 33.9 (6.2)A | 35.5 (10.3) | 41.6 (12.4)B | 40.5 (7.1) | 36.8 (5.6) |
| Waist  Circumference,  inches | 45.0 (7.3) | 44.0 (11.0) | 43.5 (4.4) | 43.0 (9.9) | 47.8 (12.3) | 47.0 (8.7) | 45.0 (3.2) |
| Systolic blood  pressure, mmHg | 127.0 (17.0) | 132.0 (19.0) | 123.5 (16.0) | 125.0 (17.0) | 133.2 (23.0) | 129.0 (22.0) | 130.5 (14.0) |
| Diastolic blood  pressure, mmHg | 76.0 (16.0) | 79.6 (19.0) | 72.5 (18.0) | 68.0 (18.0) | 80.0 (17.0) | 76.0 (8.0) | 80.5 (14.0) |
| Total cholesterol,  mg/dL | 187.5 (55.0) | 179.0 (85.0) | 187.0 (72.0) | 204.0 (47.0) | 188.0 (55.0) | 190.0 (47.0) | 174.0 (40.0) |
| LDL-cholesterol,  mg/dL | 113.0 (52.0) | 109.0 (76.0) | 96.0 (70.0) | 118.0 (52.0) | 114.0 (54.0) | 117.5 (41.00 | 102.5 (34.0) |
| HDL- cholesterol,  mg/dL | 53.0 (14.0) | 54.0 (12.0) | 52.0 (24.0) | 55.0 (23.0) | 52.5 (17.0) | 48.0 (10.00 | 54.0 (13.0) |
| Non-HDL-  cholesterol,  mg/dL | 130 (48.0) | 132.0 (83.0) | 117.0 (75.0) | 143.0 (64.0) | 134.5 (44.0) | 138.5 (49.0) | 122.0 (33.0) |
| Triglycerides,  mg/dL | 96 (57.0) | 98.0 (89.0) | 110.0 (49) | 91.5 (85.0) | 101.0 (62.0) | 92.5 (26.0) | 102.5 (67.0) |
| Glucose,  mg/dL | 90.0 (17.0) | 91.0 (13.0) | 92.0 (12.0) | 90.0 (7.0) | 84.0 (16.0) | 82.0 (27.0) | 90.0 (23.0) |
| ^1^Sample based on those that were randomized to treatment and completed the post-treatment visit (Visit 4)  2White ethnicity includes 2 Hispanic and 1 Middle Eastern participant; Black/African American ethnicity incudes 1 mixed ethnicity participant  ^3^All clinical characteristics are listed as median (IQR)  ^4^Significant difference based on Kruskal-Wallis, but no differences based on post-hoc pairwise comparisons after adjustment for multiple comparisons.  ^5^Different letters indicate significant differences between treatment groups | | | | | | | |

| Table S2: Differences in gastrointestinal symptoms by treatment group^1,2,3^ | | | | | | | |
| --- | --- | --- | --- | --- | --- | --- | --- |
|  | **Total Sample (n=92)** | **Lactose (n=16)** | **IVS-1**  **(n=14)** | **Bb12**  **(n=14)** | **IVS-1 + GOS**  **(n=15)** | **Bb12 + GOS (n=17)** | **GOS**  **(n=16)** |
| **Baseline** | |  |  |  |  |  |  |
| Bloating | 2.0 (4.8) | 2.0 (4.8) | 1.0 (3.3) | 2.0 (4.0) | 2.0 (5.0) | 4.0 (6.0) | 2.5 (7.8) |
| Passing Gas | 3.0 (5.0) | 2.5 (4.8) | 2.5 (3.5) | 2.5 (4.5) | 3.0 (5.0) | 4.0 (3.5) * | 2.5 (5.8) |
| Hard Stools | 1.0 (2.8) | 0.5 (3.0) | 1.0 (2.3) | 0.0 (2.3) | 0.0 (3.0) | 1.0 (3.5) | 1.0 (3.8) * |
| Watery Stools | 1.0 (4.8) | 1.0 (3.8) | 1.0 (3.5) | 1.5 (3.5) | 2.0 (5.0) | 1.0 (4.5) | 2.0 (5.8) |
| **Treatment End** | |  |  |  |  |  |  |
| Bloating | 1.0 (4.0) | 0.0 (4.0) | 1.5 (4.0) | 1.0 (2.0) | 1.0 (3.0) | 2.0 (4.0) | 2.5 (5.0) |
| Passing Gas | 3.0 (5.0) | 5.0 (4.8) | 3.0 (2.0) | 3.0 (6.3) | 2.0 (3.0) | 1.0 (5.0) * | 3.0 (5.8) |
| Hard Stools | 1.0 (3.0) | 0.0 (3.0) | 1.0 (1.3) | 1.0 (3.3) | 1.0 (3.0) | 0.0 (1.0)A | 3.5 (4.8)B * |
| Watery Stools | 1.0 (3.0) | 1.0 (2.0) | 1.0 (3.3) | 0.5 (1.5) | 1.0 (4.0) | 1.0 (2.5) | 2.5 (4.8) |
| ^1^Per-protocol sample based on those that were randomized to treatment and were considered compliant to the treatment | | | | | | | |
| ^2^Change in gastrointestinal symptoms are listed as median (IQR). Only the most common symptoms experienced are listed. Symptoms were recorded on a Likert scale ranging from 0-10. | | | | | | | |
| ^3^Different letters indicate a significant difference in distribution between groups within symptom; identical symbol indicate differences in symptoms before and after treatment within a treatment group | | | | | | | |

| Table S3: Percent change in intestinal permeability in subjects by treatment group^1,2^ | | | | | | | |
| --- | --- | --- | --- | --- | --- | --- | --- |
|  | **Total Sample (n=94)** | **Control (n=17)** | **IVS-1 (n=14)** | **Bb12 (n=14)** | **IVS-1+GOS (n=16)** | **Bb12+GOS (n=17)** | **GOS**  **(n=16)** |
| 5-hour Mannitol^3^ |  |  |  |  |  |  |  |
| % change no  aspirin * | -21.0 (87.1) | -50.4 (104.3) | -47.8 (65.6) | -27.2 (56.9) | 55.6 (600.7) | -11.9 (99.5) | -21.9 (169.5) |
| % change aspirin | -19.9 (128.0) | -5.6 (585.3) | -40.2 (154.7) | -41.8 (44.5) | -8.0 (1149.6) | -40.8 (161.0) | -2.2 (47.5) |
| 5-hour Sucrose^4^ |  |  |  |  |  |  |  |
| % change no  aspirin | -0.2 (84.8) | -27.8 (80.1) | 3.3 (32.7) | -10.1 (97.5) | -1.1 (208.3) | -19.9 (93.8) | 36.1 (63.2) |
| % change aspirin | -14.3 (114.7) | -10.5 (106.9) | -7.8 (183.9) | -31.7 (78.4) | -20.9 (128.1) | -17.5 (152.7) | -16.9 (92.3) |
| 24-hour Sucralose^5^ |  |  |  |  |  |  |  |
| % change no  aspirin | -15.1 (80.6) | -16.1 (63.8) | -35.4 (130.3) | -29.2 (69.0) | -14.1 (96.9) | 23.0 (61.4) | -26.7 (52.8) |
| % change aspirin | -26.1 (68.9) | 37.2 (152.9) | -30.8 (92.7) | -25.8 (9.8) | -18.3 (138.8) | -12.7 (69.8) | -30.5 (33.28) |
| 24-hour Sucralose:lactulose^6^ | | | | | | | |
| % change no  aspirin | -11.3 (94.4) | -14.4 (91.2) | -35.3 (99.5) | -32.4 (57.9) | -8.0 (233.8) | 2.6 (324.3) | -18.1 (120.4) |
| % change aspirin | -27.6 (69.3) | 6.8 (145.3) | -55.5 (76.9) | -27.8 (54.5) | -27.6 (125.0) | -9.7 (54.5) | -45.6 (57.4) |
| ^1^Per-protocol sample based on those that were randomized to treatment and were considered compliant to the treatment  ^2^All intestinal permeability measurements are listed as median (IQR)  ^3^n= 81 and 68 for percent change with and without aspirin, respectively  ^4^n=83 and 77 for percent change with and without aspirin, respectively  ^5^n= 79 and 69 for percent change with and without aspirin, respectively  ^6^n= 80 and 69 for percent change with and without aspirin, respectively  ^*^difference in the distribution of percent change across treatment groups (*P*=0.036); no significant differences in post-hoc pairwise comparisons between groups | | | | | | | |

| Table S5: Percent change in anthropometrics and metabolic markers in subjects by treatment group^1,2,3^ | | | | | | | | | | | |
| --- | --- | --- | --- | --- | --- | --- | --- | --- | --- | --- | --- |
|  | **Total**  **Sample**  **(n=94)** | **Lactose**  **(n=17)** | | **IVS-1**  **(n=14)** | **Bb12**  **(n=14)** | | **IVS-1 + GOS**  **(n=16)** | | **Bb12 + GOS**  **(n=17)** | **GOS**  **(n=16)** | |
| **Anthropometrics** | |  | |  |  | |  | |  |  | |
| Body  weight,  kg | 0.4 (2.9) | 0.4 (5.0) | | -0.1 (2.9) | 0.6 (2.4) | | -0.3 (3.4) | | -0.1 (3.6) | 1.3 (3.6) | |
| BMI,  kg/m^2^ | 0.4 (2.9) | 0.4 (5.0) | | -0.1 (2.9) | 0.6 (2.4) | | -0.3 (3.4) | | -0.1 (3.6) | 1.3 (3.6) | |
| Waist  circum-  ference,  inches | 0.0 (4.6) | -1.3 (3.8) | | 0.0 (6.9) | 0.2 (5.2) | | 0.0 (5.5) | | 1.8 (3.0) | 0.0 (5.1) | |
| **Blood pressure** | |  | |  |  | |  | |  |  | |
| Systolic,  mmHg | -0.4 (14.4) | 2.6 (16.0) | | -6.6 (13.5) | 1.4 (12.3) | | -0.4 (19.8) | | -0.8 (18.4) | -2.0 (10.7) | |
| Diastolic,  mmHg | -2.4 (14.3) | 0.0 (16.4) | | -8.2 (24.3) | -2.3 (15.5) | | -5.7 (11.7) | | -2.0 (12.5) | 0.6 (14.8) | |
| **Serum lipids** |  |  | |  |  | |  | |  |  | |
| Total choles-  terol, mg/dL | -2.5 (12.1) | -1.9 (10.0) | | -2.7 (14.6) | -8.8 (17.4) | | -7.0 (9.5) | | -0.5 (9.2) | 1.9 (13.9) | |
| LDL-choles-  terol, mg/dL | -3.3 (15.8) | -1.3 (17.0) | -1.3 (20.3) | | | -7.6 (21.3) | -7.5 (9.8) | 2.2 (11.8) | | | -1.7 (17.6) |
| HDL- choles-  terol, mg/dL^4^ | -1.0 (13.4) | -3.1 (12.1) | -3.2 (9.8) | | | -3.8 (13.2)A | -5.4 (11.1) | 3.7 (15.9)B | | | -1.9 (17.0) |
| Non-HDL-  cholesterol,  mg/dL | -5.8 (13.2) | -3.4 (12.0) | -8.6 (16.5) | | | -8.7 (17.3) | -6.7 (7.4) | -0.5 (12.5) | | | -1.3 (17.6) |
| Triglycerides,  mg/dL | -9.9 (26.0) | -16.6 (37.1) | 0.0 (18.7) | | | -13.4 (31.30 | -5.6 (33.1) | -8.7 (28.0) | | | -6.7 (24.5) |
| **Glucose metabolism** | |  |  | | |  |  |  | | |  |
| Glucose,  mg/dL | -2.4 (15.9) | -4.0 (16.6) | -6.0 (14.7) | | | -4.0 (23.7) | 1.4 (21.0) | -0.7 (20.7) | | | -2.4 (12.1) |

^1^Per-Protocol sample based on those that were randomized to treatment and were considered compliant to the treatment

^2^All clinical characteristics are listed as median (IQR)

^3^n=92 for glucose, blood pressure, waist circumference, body weight, and BMI; n=91 for LDL-Cholesterol and Non-HDL-Cholesterol; n=89 for HDL-Cholesterol and triglycerides

^4^significant differences based on Kruskal-Wallis; significant difference between Bb12 and Bb12+GOS by pairwise comparison, P=0.032
