# Supplementary figures and images for: Probiotic Bifidobacterium strains and galactooligosaccharides improve intestinal barrier function in obese adults but show no synergism when used together as synbiotics
Source: Microbiome. 2018 Jun 28;6:121. doi: 10.1186/s40168-018-0494-4 (PMC6022452; doi:10.1186/s40168-018-0494-4)

## Slide 1
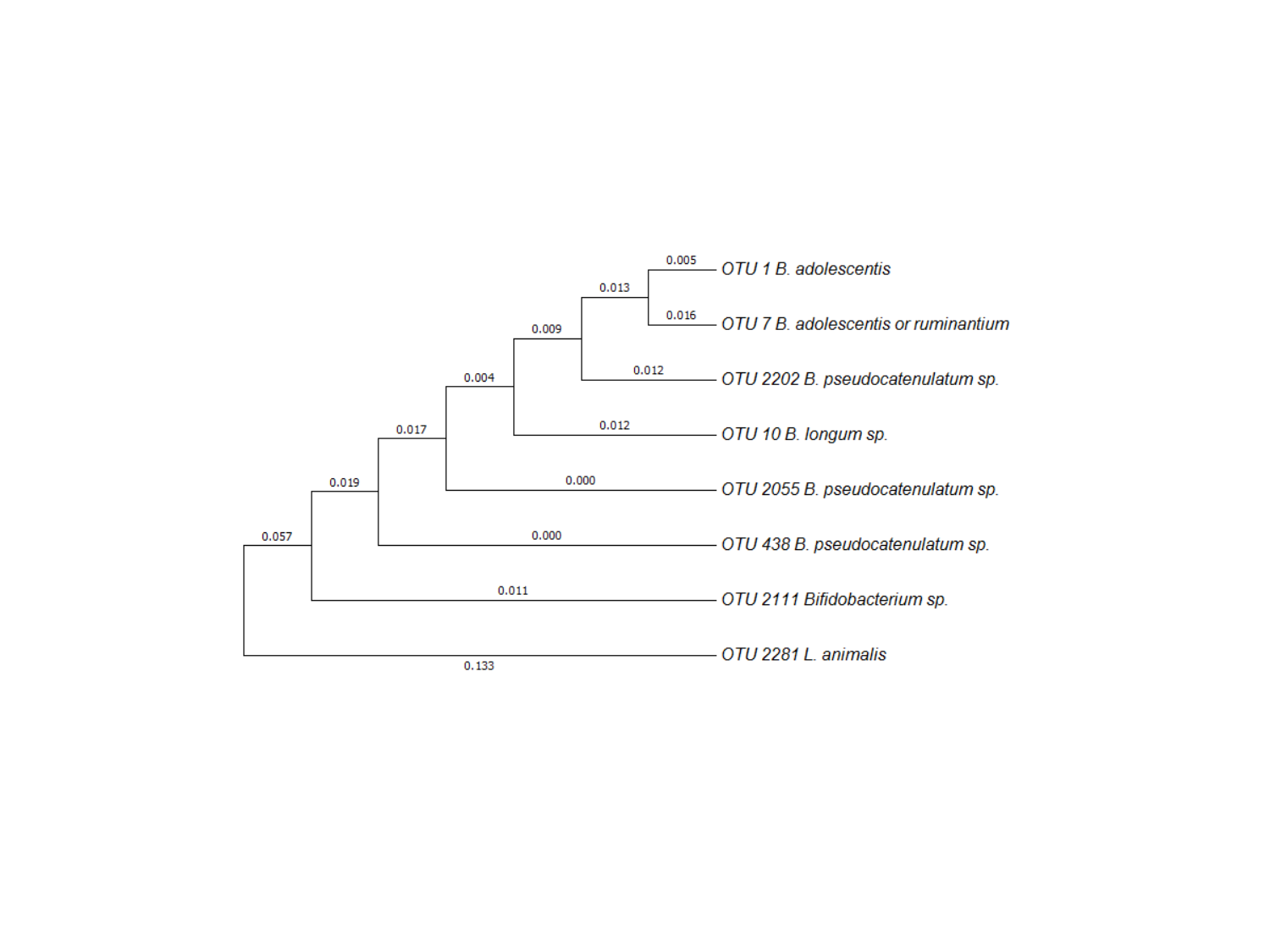

Supplement: Supplementary file 2 — Figure S1. Phylogenetic analysis of OTU_1 and six closely related Bifidobacterium OTUs that might have competed with OTU_1 for the niche in the GI tract using Maximum Likelihood method. OTU_2281 Lactobacillus animalis was selected as the Outgroup. Numbers indicate branch lengths. (PPTX 41 kb) [file 40168_2018_494_MOESM2_ESM.pptx]
